# Development of a patient satisfaction questionnaire (PSQ) for diabetes management in Thailand and Lao PDR

**DOI:** 10.1371/journal.pone.0300052

**Published:** 2024-03-07

**Authors:** Phayom Sookaneknun Olson, Chanuttha Ploylearmsang, Phoutsathaphone Sibounheuang, Santiparp Sookaneknun, Chanthanom Manithip, Suntaree Watcharadamrongkun, Paul W. Jungnickel, Pattarin Kittiboonyakun

**Affiliations:** 1 Faculty of Pharmacy, International Primary Care Practice Research Unit, Mahasarakham University, Khamriang Sub-District, Kantarawichai District, Maha Sarakham, Thailand; 2 Faculty of Pharmacy, University of Health Sciences, Kao Ngot Villagem, Sisattanak District, Vientiane Capital, Lao PDR; 3 Mahasarakham Business School, Mahasarakham University, Khamriang Sub-District, Kantarawichai District, Maha Sarakham, Thailand; 4 Ministry of Health, Ban Thatkhao, Sisattanack District, Rue Simeuang, Lao PDR; 5 Faculty of Pharmaceutical Sciences, Chulalongkorn University, Bangkok, Thailand; 6 Department of Pharmacy Practice, Harrison College of Pharmacy, Auburn University, Auburn, Alabama, United States of America; 7 Faculty of Pharmacy, Health Service and Pharmacy Practice Research and Innovation Unit, Mahasarakham University, Khamriang Sub-District, Kantarawichai District, Maha Sarakham, Thailand; University of South Australia, AUSTRALIA

## Abstract

In a cross-sectional analytical study, a Patient Satisfaction Questionnaire (PSQ) for diabetes management was developed and tested in Thailand and Lao PDR. A systematic review of qualitative studies was conducted to formulate themes of the PSQ. The 20-item PSQ was prepared in Thai and translated to Lao, with subsequent backward translation. Both versions were tested for reliability and construct validity using confirmatory factor analysis and structural equation modeling. The study was performed at a university hospital in Thailand and two central hospitals in Vientiane, Lao PDR. There were 300 diabetic patients from Thailand (n = 150) and Lao PDR (n = 150). The 5-factor Thai version showed 74.52% of total explained variance with good internal consistency and satisfactory goodness-of-fit indices (χ2/df = 1.91, GFI = 0.83, CFI = 0.98, SRMR = 0.063, RMSEA = 0.078). The five factors were 1) Standard of Service, 2) Diabetic Service, 3) Competency of Providers, 4) Competency of Pharmacists, and 5) Communication with Providers. For the Lao version, 20 items showed a 3-factor structure with a total explained variance of 71.09%. Goodness-of-fit indices for the Lao model were satisfactory (χ2/df = 2.45, GFI = 0.78, CFI = 0.95, SRMR = 0.075 and RMSEA = 0.095). The results showed the PSQ Thai and Lao versions were valid and reliable for assessing patient satisfaction with diabetes management, however more testing of the questionnaire is appropriate.

## Introduction

Diabetes is a major health issue that has reached alarming levels. In 2021, an estimated 537 million adults aged 20–79 years (10.5% of all adults in this age group) had diabetes. By 2045, 783 million adults aged 20–79 years (12.2% of all adults in this age group) are projected to be living with diabetes [1]. It is estimated that over 6.7 million people aged 20–79 years will die from diabetes-related causes in 2021. This corresponds to 12.2% of global deaths from all causes in this age group [2]. The estimated prevalence of diabetes in Lao People’s Democratic Republic (Lao PDR) is 6.2% (214,800 diabetes adults aged 20–79 years) and is projected to be 7.5% in 2045 [3]. In Thailand, the estimated prevalence of diabetes in adults is 9.7%; 6 million adults aged 20–79 years have diabetes and the incidence is projected to be 11.0% by 2045 [3].

One systematic review on diabetes management addressed barriers, such as limited accessibility to care, lack of coordination in referral systems, poor interaction between patients and health providers, and lack of communication skills. The study also encouraged providers’ support, family involvement and community resources for diabetes patients [4].

Lao PDR and Thailand have similar cultures and languages. However, their healthcare systems are totally different. Lao PDR has only four central hospitals located in the capital city, with each hospital providing diabetes care services [5]. Although hospital diabetes care service in Lao PDR consists of an inter-disciplinary team including endocrinology physicians, nurses, and pharmacists, the main role of the hospital pharmacist is to dispense medications. The national health insurance (NHI) coverage in Lao PDR has been increasing and covered 94% of the population in 2018 [6], though some patients still have to pay from their pockets for diabetes treatment because the NHI provides payment for services at a flat rate and patients are expected to pay 25% of surgery costs that exceed 600 USD [7]. In Thailand, universal health care covers 75% of the total population (the rest of the population is covered by the civil servant scheme or the social security scheme) and provides a comprehensive benefits package with zero co-payment at the point-of-services [8]. Thailand has its own guidelines for diabetes treatment. The Ministry of Public Health endorsed the “Thailand Healthy Lifestyle Strategy 2011–2020 plan” to reduce prevalence, complications, disability, mortality, and cost of illness in diabetes. However, diabetes management in Thailand still faces many challenges such as access to treatment, inequalities, high healthcare worker turnover in rural areas, and low public awareness of diabetes issues especially among less well-educated people [9].

Patient satisfaction is an important indicator reflecting the quality of a service [10]. Some studies assessed satisfaction as the primary outcomes [11]. Satisfaction can be assessed from several perspectives, such as those of healthcare providers and patients. Perspectives from patients are important indicators which reflect quality of service [10,12]. One systematic review included several studies of patient satisfaction questionnaires that were developed and tested for validity and reliability [13,14]. Questionnaires have been used to evaluate patient satisfaction with various dimensions of care. Anderson et al developed the 22-item insulin treatment satisfaction questionnaire, however this tool does not address satisfaction with noninsulin medications [15]. Paddock et al constructed a 73-item questionnaire to evaluate patient satisfaction with diabetes disease management programs (DDMPs) provided by teams of health care professionals [16]. While useful, the results of this study may be limited to structured DDMPs, but not routine practices of diabetes management in other settings. Brose and Bradley developed the 13-item satisfaction questionnaire for retinopathy treatment from patient interviews. This questionnaire is a specific quality of life measure for patients with diabetic retinopathy [17]. Wilbur et al tested the 8-item diabetes treatment satisfaction questionnaire (DTSQ) in an Arabic version. The DTSQ evaluated treatment satisfaction including current treatment, treatment convenience, flexibility of treatment, understanding diabetes, continuity of treatment, and recommending treatment to other diabetic patients [18]. Franciosi et al reported satisfactory findings using the American Board of Internal Medicine 14 patient satisfaction questionnaire that explores patient satisfaction regarding physicians’ humanness and communication skills [19].

While there are a variety of tools available, limitations in the assessment of the quality of diabetes care in clinical settings remain [20]. Although available tools have unique strengths, they do not included measures of access to health care and provide limited measurement of relationships between providers and patients. There are no instruments that assess diabetes management in routine practice with pharmacists’ involvement. Thus, this study aimed to develop a satisfaction questionnaire from the perspective of diabetic patients to assess diabetes care services in Thailand and Lao PDR.

## Materials and methods

### Design

This study developed and validated a questionnaire. The methods and tools (S1 File) for this study were approved by the Mahasarakham University Ethical Board for Human, approval number 063/2561, and Lao National Ethics Committee for Health Research, approval number 081/NECHR. Date: 22 Aug 2018. Written informed consent was obtained from eligible participants who participated in the study.

### Questionnaire dimensions and items formulation

The dimensions and items of the patient satisfaction questionnaire (PSQ) were constructed from main themes and sub-themes from systematic review of qualitative studies of patients’ and healthcare providers’ perspectives on diabetes management [4]. The PSQ consists of 20 items in five dimensions of satisfaction which are 1) Standard of Service, 2) Diabetes Service, 3) Competency of Providers, 4) Competency of Pharmacists, and 5) Communication with Providers. Each dimension consists of four items.

Content validity was evaluated by four experts: two were Ph.Ds. in clinical pharmacy, one was a Ph.D. in social and administrative pharmacy, and one was Ph.D. in questionnaire development. All the items were scored on 5-point Likert scale responses: 1 (“not at all satisfied”), 2 (“slightly satisfied”), 3 (“moderately satisfied”), 4 (“very satisfied”), and 5 (“completely satisfied”). In the event that the patients had never received specific services, they did not have to answer these items. Complete-case analysis was used for missing data management. Reliability testing of the Thai version was undertaken in 30 participants from a community hospital in Thailand (Cronbach alpha = 0.940).

### Translation process

The Thai and Lao languages are similar in listening, but not writing. The interpreters involved in the translation process used both Thai and Lao languages in their work and daily life and were defined as having actual language proficiency [21]. The procedures of translation from the Thai language to the Lao language were as follows: 1) Translation was done by a Lao translator who could read Thai fluently; the questionnaire in Lao version was issued (PSQ Lao1), 2) A discussion by a member of the research team and the Lao translator was held to compare the Lao version, PSQ Lao1, with the Thai version to make adjustments, PSQ Lao2, 3) The Lao version, PSQ Lao2, was translated back into the Thai language by another Lao translator who could read Thai fluently, 4) The Lao questionnaire from step 3 was compared with the Thai language version to determine any differences and make adjustments between a member of the research team and the second Lao translator; PSQ Lao3 was issued and was the final version, and 5) The PSQ Lao3 final version was tested for feasibility and reliability in 30 diabetic participants in Lao PDR (Cronbach alpha = 0.925).

### Participants

Participants represented a convenience sample and were recruited from a university hospital in Thailand and two central hospitals in Vientiane, Lao PDR. Inclusion criteria were patients with diabetes who were aged ≥ 18 years, diagnosed with type 2 diabetes mellitus (T2DM), treated in the study hospitals, and willing to participate the study. Participants were excluded if they did not receive medications for diabetes treatment. According to Hair et al. (2014), the minimum sample size for this type of study should be at least five times the number of variables to be analyzed [22]. For the 20-item PSQ with 5 dimensions in this study, there were 20 variables to be analyzed. Thus, the minimum sample should be 100 (20 multiplied by 5). The 150 subjects from each country exceeded the minimum target sample size needed and represented more observations than variables for confirmatory factor analysis (CFA). The study was undertaken during November 2018 in Thailand, and during December 2018 and January 2019 in Lao PDR.

### Statistical methods

Demographic variables were summarized as means, standard deviations, and proportions. For comparisons of continuous variables, independent t-tests and Mann-Whitney U tests were utilized depending on the normality distribution of the data. For the comparisons of proportions in demographic variables, Chi-square tests were applied.

The exploratory data analysis and reliability analysis were carried out via SPSS. LISREL 8.33 (Linear Structural Relation Statistics Package Program) was used for confirmatory factor analysis (CFA) and structural equation modeling (SEM). To assess construct validity and determine whether applying factor analysis to the set of data was suitable, the Kaiser–Meyer–Olkin (KMO) and Bartlett Sphericity tests were conducted. The appropriateness criteria of conducting CFA are ≥ 0.5 for the KMO test and *p* < 0.001 for Bartlett’s test of sphericity [22]. For scale evaluation, Cronbach’s alpha for reliability and average variance explained for validity were computed; Cronbach’s alpha >0.7 was considered acceptable [22].

Principal Component Analysis was performed on each set of data; this gives eigenvalues (>1.00). Varimax rotation was applied in the analysis. Items on the PSQ were divided into factors based on the factor loadings. All items were assigned to a factor if its loading was greater than 0.40 [23]. The fit of CFA model was evaluated using the following fit indices: model chi-square (χ^2^), the ratio of chi-square to its degrees of freedom (χ^2^/df), goodness fit index (GFI), comparative fit index (CFI), standardized root means square residual (SRMR), and root mean square error of approximation (RMSEA). Model Fit Statistics from LISREL, such as χ2 /df <5, GFI ≥0.80, CFI≥0.90, and RMSEA and SRMR <0.08 were the criteria to evaluate the results of CFA and SEM [24]. Differential item functioning (DIF) analysis was applied to assess the bias between the Thai and Lao versions [25]. An intraclass correlation coefficient (ICC) >0.5 was used [26].

## Results

A total of 300 participants with diabetes completed the study. The demographic data for the participants is presented in Table 1. Five characteristics were significantly different between Thai and Lao participants: income, number of family members, distance from diabetic services, co-morbidities, and insurance (p<0.05).

**Table 1:** Demographic data of Lao and Thai diabetes patients.

Characteristics	Lao PDR (n = 150)	Thailand (n = 150)	p-value
	Number (%)	Number (%)	
Sex: male	71 (47.3)	88 (58.7)	0.064 ^a^
Age (year) (mean±SD)	56.8 ± 10.82	60.7 ± 11.27	0.685 ^b^
Occupation Civil servant (Government Officer) Employee Commercial Farmer Retired Other (no job)	37 (24.7) 14 (9.3) 19 (12.7) 3 (2.0) 32 (21.3) 45 (30.0)	38 (25.3) 13 (8.7) 28 (18.7) 18 (12.0) 30 (20.0) 23 (15.3)	0.099 ^a^
Education No education Elementary Primary school High school Diploma Bachelor Higher than bachelor	6 (4.0) 35 (23.5) 31 (20.8) 25 (16.8) 16 (10.7) 24 (16.1) 12 (8.1)	9 (6.0) 34 (22.7) 14 (9.3) 26 (17.3) 9 (6.0) 38 (25.3) 20 (13.3)	0.091 ^a^
Income per month (n _Lao_ = 149) ≤ 143 USD 143–285.7 USD 285.8–428.6 USD 428.7–571.4 USD ≥ 571.5 USD	60 (40.3) 56 (37.6) 23 (15.4) 10 (6.7) n/a	46 (30.7) 23 (15.3) 13 (8.7) 12 (8) 56 (37.3)	<0.001 ^a^
Married status (n _Lao_ = 149) Single Married Widow Divorced Separated	6 (4.0) 121 (81.2) 18 (12.1) 3 (2.0) 1 (0.7)	10 (6.7) 103 (68.7) 26 (17.3) 7 (4.6) 4 (2.7)	0.082 ^a^
Family members (number) (mean±SD)	4.82 ± 2.43	3.85 ± 1.77	<0.001 ^c^
Distance from health service (kilometers) (mean±SD)	23.48 ± 89.82	8.67 ± 12.7	<0.007 ^c^
Duration of diabetes (years) (mean±SD)	8.29 ± 7.21	8.83 ± 7.58	0.861 ^c^
Co-morbidity: Yes	79 (53.4)	112 (74.7)	<0.001 ^a^
Source of media for diabetes knowledge Healthcare providers Radio/TV Social media Friends/cousins Various sources	98 (66.7) 9 (6.1) 1 (0.7) 10 (6.8) 28 (19.7)	93 (62.0) 7 (4.7) 3 (2.0) 5 (3.3) 42 (28)	0.139 ^a^
Health insurance Universal coverage Civil servant medical benefit scheme Social security Community insurance Private insurance Out of pocket	3 (2.0) 78 (52.7) 27 (18.2) 7 (4.7) 1 (0.7) 32 (21.6)	48 (32.0) 2 (1.3) 88 (58.7) 10 (6.7) 1 (0.7) 1 (0.7)	<0.001 ^a^

^a^ Chi-square test

^b^ Independent t-test

^c^ Mann-Whitney U Test.

SD stands for standard deviation.

**Fig 1:**
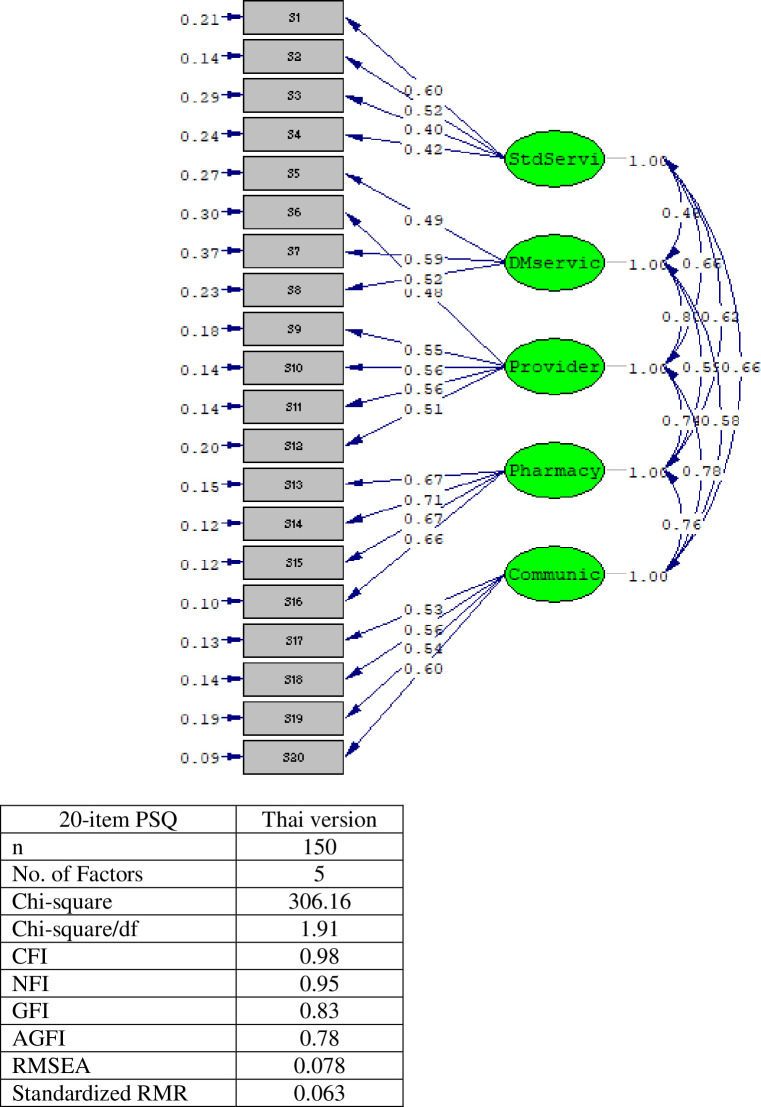
Model of 20-item PSQ Thai version.

Using the sample of the 150 Thai patients, the CFA model for the PSQ Thai version showed five factors of Patient Satisfaction that explained 74.52% of total variance (Table 2). The lowest mean was in item S7-satisfaction with home care services (3.42±0.85), and the highest mean was in item S4-satisfaction with insurance (4.34±0.64). The 5 dimensions of the PSQ were 1) Standard of Service, 2) Diabetes Service, 3) Competency of Providers, 4) Competency of Pharmacists, 5) Communication with Providers. As shown in Table 1, all factor loadings were acceptable (range = 0.478–0.853) with significant meaning for the model, indicating that scale items on the PSQ Thai version are of high measurement quality. In the Diabetes Service dimension, item 6 loaded on the Competency of Providers dimension, so was included as part of this factor. The ICC was 0.907 (95% CI 0.884–0.927). The CFA model of the PSQ Thai version had satisfactory goodness-of-fit on most fit indices (χ^2^_(149)_ = 306.16, *p* < 0.001, CFI = 0.98, GFI = 0.83, SRMR = 0.063, RMSEA = 0.078) (Table 2 and Fig 1).

**Table 2:** Factor loading of 20 items of patient satisfaction (Thai version).

Dimensions and Items	Factor 1	Factor 2	Factor 3	Factor 4	Factor 5	h^2^	Mean	SD
**Dimension 1: Standard of Services**								
S1-satisfaction with a separate diabetes service from others	.703					.700	4.20	.75
S2-satisfaction with continuing health check up	.781					.782	4.25	.64
S3- satisfaction with annual health check ups	.640					.663	4.03	.67
S4-satisfaction with health insurance	.707					.606	4.34	.64
**Dimension 2: Diabetes Service**								
S5-satisfaction with diabetes education services		.592				.657	4.09	.71
S6-satisfaction with diabetes follow-up services			.615			.651	4.13	.73
S7-satisfaction with home care service		.833				.773	3.42	.85
S8-satisfaction with the evaluation system of the service unit (e.g., satisfaction questionnaire to the service)		.630				.661	3.77	.71
**Dimension 3: Competency of Providers**								
S9-satisfaction with the understanding of medical history			.561			.661	4.05	.69
S10-satisfaction with usefulness of information			.747			.790	4.13	.67
S11-satisfaction with specific individual plan			.720			.753	4.04	.67
S12-satisfaction with the amount of service time given by the healthcare providers			.478			.670	4.02	.68
**Dimension 4: Competency of Pharmacists**								
S13-satisfaction with the pharmacist regarding medication history				.853		.866	4.05	.77
S14-satisfaction with the usefulness of information on diabetic medication by the pharmacist				.819		.871	4.06	.79
S15-satisfaction with the medication plan of the pharmacist				.739		.822	4.07	.75
S16-satisfaction with the service time given by the pharmacist				.771		.833	4.03	.74
**Dimension 5: Communication with Providers**								
S17-satisfaction with the communications with healthcare providers					.802	.785	4.18	.65
S18-satisfaction with the service mindedness of the healthcare providers					.736	.785	4.25	.67
S19-satisfaction with the coordination of healthcare among the healthcare providers					.733	.762	4.04	.69
S20-satisfaction with the language of communications					.750	.814	4.14	.68
**Factor names**	**1.Standard of Service**	**2.Diabetic Service**	**3.Competency of Providers**		**4.Competency of Pharmacists**		**5.Communication with Providers**	
No. of item	S1-S4	S5, S7-S8	S6, S9-S12		S13-S16		S17-S20	
% Variances	14.612	10.825	14.387		16.813		17.876	
Cronbach’s Alpha Coefficients (α)	.801	.746	.876		.907		.939	
Average Variance Extracted	.305	.412	.368		.960		.306	
Composite Reliability (Omega coefficient)	.715	.720	.832		.974		.787	
% Total Explained Variance (R^2^)	74.522							

^**a**^ Factor loading after adjusted (cut the items that were lower than 0.4), Extraction Method: Principal Component Analysis. Rotation Method: Varimax with Kaiser Normalization.

KMO (Kaiser-Meyer-Olkin Measure of Sampling Adequacy) = 0.920, Bartlett’s Test of Sphericity (p<0.001).

**Fig 2:**
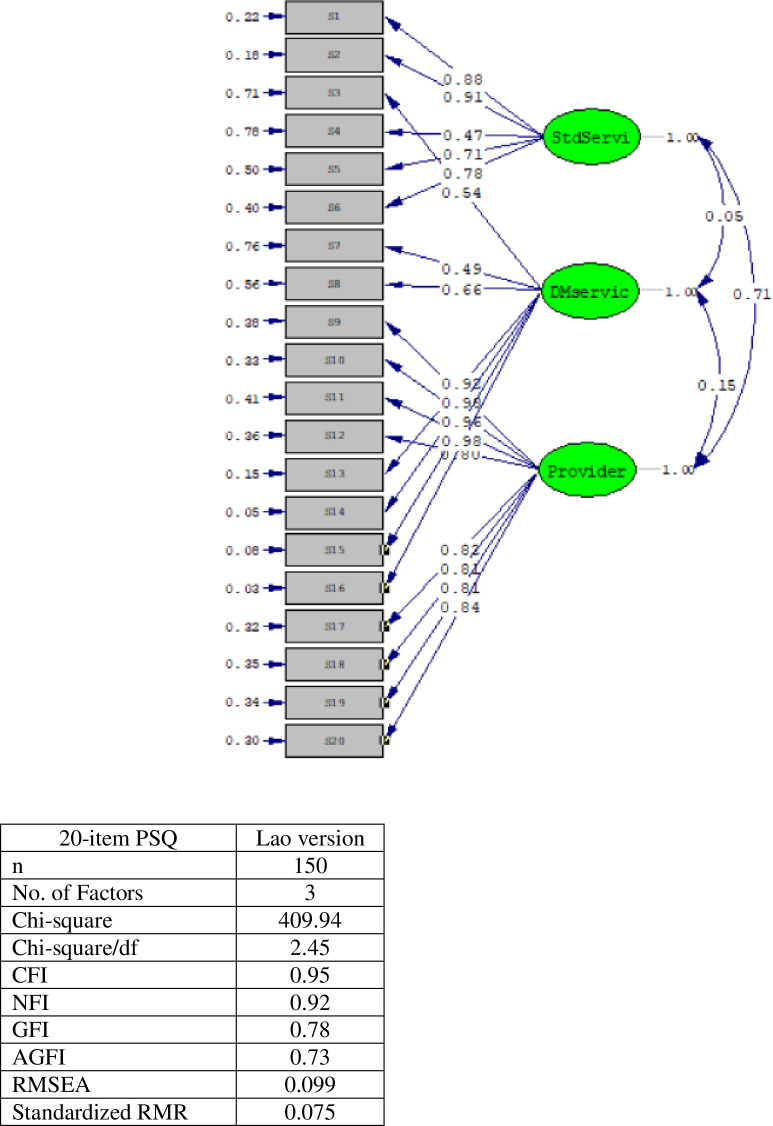
Model of 20-item PSQ Lao version.

The PSQ Lao version was tested in 150 Lao patients. As shown in Table 3, all factor loadings were acceptable (range = 0.503–0.866). The lowest mean was in item S7-satisfaction with home care service (3.12±0.75), and the highest mean was in item S2-satisfaction with continuing health checkup (4.22±0.91). The CFA model showed three factors that explained 71.09% of total variance. The first factor, Standard of Services was derived from dimension 1 (S1,2,4) and dimension 2 (S5-6). The second factor, Diabetes Service, was derived from a combination of dimension 1 (S3), dimension 2 (S7-8), and dimension 4 (S13-16). And the third factor, Satisfaction with Providers, was derived from a combination of dimension 3 (S9, S12) and dimension 5 (S17-20) as shown in Fig 2. The ICC was 0.968 (95% CI 0.955–0.980). Model chi-square (χ^2^) = 409.94, the ratio of chi-square to its degrees of freedom (χ^2^/df) = 2.45, GFI = 0.78, CFI = 0.95, and SRMR and RMSEA (0.075 and 0.095 respectively), were satisfactory (Table 3, Fig 2).

**Table 3:** Factor loading of 20 items of patient satisfaction (Lao version).

Dimensions and items	Factor 1	Factor 2	Factor 3	h^2^	Mean	SD
**Dimension 1: Standard of Services**						
S1-satisfaction with a separate diabetes service from others	.854			.788	4.13	.96
S2-satisfaction with the continuing health check up	.845			.779	4.22	.91
S3- satisfaction with the annual health check up	.655			.580	3.72	1.24
S4-satisfaction with health insurance	.672			.718	4.02	1.06
**Dimension 2: Diabetes services**						
S5-satisfaction with diabetes education services	.746			.730	4.02	.92
S6-satisfaction with diabetes follow-up services	.757			.716	4.17	.91
S7-satisfaction with home care service		.504		.374	3.12	.75
S8-satisfaction with the evaluation system of the service unit (e.g., satisfaction questionnaire to the service)		.720		.721	3.63	.94
**Dimension 3: Competency of Providers**						
S9-satisfaction with the understanding of medical history	.503			.646	3.93	.93
S10-satisfaction with the usefulness of information			.582	.658	4.07	.92
S11-satisfaction with specific individual plan			.619	.797	4.03	.95
S12-satisfaction with the amount of service time given by the healthcare providers			.644	.744	4.06	.86
**Dimension 4: Competency of Pharmacists**						
S13-satisfaction with the pharmacist regarding medication history		.644		.660	3.82	.86
S14-satisfaction with the usefulness of information on diabetic medication by the pharmacist		.650		.763	3.93	.89
S15-satisfaction with the medication plan of the pharmacist		.724		.731	3.95	.92
S16-satisfaction with the service time given by the pharmacist			.645	.805	3.91	.93
**Dimension 5: Communication with Providers**						
S17-satisfaction with the communications with healthcare providers			.832	.791	4.07	.87
S18-satisfaction with the service mindedness of the healthcare providers			.866	.816	4.08	.84
S19-satisfaction with the coordination of healthcare among the healthcare providers			.630	.650	4.05	.81
S20-satisfaction with the language of communications			.744	.751	4.13	.81
**Factor names**	**1.Standard of Service**		**2.Diabetic Service**		**3.Satisfaction with Providers**	
No. of item	S1-S6, S9		S7, S8, S10-S15		S16-S20	
% Variances	56.767		9.110		5.213	
Cronbach’s Alpha Coefficients (α)	.921		.910		.927	
Average Variance Extracted	.591		.545		.552	
Composite Reliability (Omega coefficient)	.768		.894		.711	
% Total Explained Variance (R^2^)	71.091					

^**a**^ Factor loading after adjusted (cut the items that were lower than 0.4) Extraction Method: Principal Component Analysis.

Rotation Method: Varimax with Kaiser Normalization.

KMO (Kaiser-Meyer-Olkin Measure of Sampling Adequacy) = 0.918. Bartlett’s Test of Sphericity (p<0.001).

The differential item functioning, or DIF, was analyzed to test items on which the Thai and Lao subject groups performed differently using the Mantel-Haenszel χ2 test. The MHχ2 of 20 items between the two nations’ samples were in the range of 0.335–14.387 and showed significant differences for 10 items (S2-4, S7-11, and S15-16) as shown in Table 4. This might be one of the reasons for the difference between the Thai and Lao PSQ models.

**Table 4:** Comparison between Thai and Lao versions by using beta, standard beta, t-test, and Mantel-Haenszel χ^2^ test.

Dimensions and items	Beta		Standard beta		t-test		MHχ^2^
	Thai	Lao	Thai	Lao	Thai	Lao	
**Dimension 1: Standard of Services**							
S1-satisfaction with a separate diabetes service from others	0.60	0.90	0.80	0.88	10.86	13.39	0.779
S2-satisfaction with the continuing health check up	0.52	0.88	0.81	0.91	11.09	14.00	5.053*
S3- satisfaction with the annual health check up	0.40	0.83	0.60	0.47	7.51	5.80	14.387*
S4-satisfaction with health insurance	0.42	0.79	0.65	0.71	8.23	9.64	14.219*
**Dimension 2: Diabetes services**							
S5-satisfaction with diabetes education services	0.49	0.79	0.68	0.78	8.53	11.00	1.650
S6-satisfaction with diabetes follow-up services	0.59	1.08	0.70	0.54	8.79	7.12	2.200
S7-satisfaction with home care service	0.52	0.76	0.74	0.49	9.45	6.30	4.151*
S8-satisfaction with the evaluation system of the service unit (e.g., satisfaction questionnaire to the service)	0.48	1.31	0.66	0.66	8.80	9.08	7.554*
**Dimension 3: Competency of Providers**							
S9-satisfaction with the understanding of medical history	0.55	1.90	0.79	0.90	11.30	14.87	7.201*
S10-satisfaction with the usefulness of information	0.56	2.06	0.83	0.96	12.20	16.42	4.153*
S11-satisfaction with specific individual plan	0.56	2.04	0.83	0.96	12.07	15.84	5.152*
S12-satisfaction with the amount of service time given by the healthcare providers	0.51	2.09	0.75	0.98	10.42	16.72	1.562
**Dimension 4: Competency of Pharmacists**							
S13-satisfaction with the pharmacist regarding medication history	0.67	0.84	0.87	0.78	13.23	11.28	2.011
S14-satisfaction with the usefulness of information on diabetic medication by the pharmacist	0.71	0.84	0.90	0.82	14.09	11.99	1.999
S15-satisfaction with the medication plan of the pharmacist	0.67	0.85	0.89	0.77	13.79	10.98	5.388*
S16-satisfaction with the service time given by the pharmacist	0.66	0.78	0.90	0.80	14.07	11.57	3.893*
**Dimension 5: Communication with Providers**							
S17-satisfaction with the communications with healthcare providers	0.53	0.81	0.83	0.82	12.06	12.16	3.429
S18-satisfaction with the service mindedness of the healthcare providers	0.56	0.73	0.83	0.81	12.27	11.81	2.612
S19-satisfaction with the coordination of healthcare among the healthcare providers	0.54	0.80	0.78	0.81	11.12	11.92	0.335
S20-satisfaction with the language of communications	0.60	0.73	0.89	0.84	13.60	12.42	1.173

* p value < 0.05.

## Discussion

This study presented a new tool for measuring patient satisfaction with diabetes management in Thailand and Lao PDR, which was developed in the Thai and Lao languages using forward and back translation. The 20-item questionnaire included five dimensions: 1) Standard of Service, 2) Diabetes Service, 3) Competency of Providers, 4) Competency of Pharmacists, 5) Communication with Providers. Reliability test results were high for both versions (Cronbach alpha>0.7). CFA demonstrated the validity of the PSQ Thai version with five factors and the PSQ Lao version with three factors.

The strength and distinctive feature of the PSQ used in this study, is that it was developed as a generic tool to assess patients’ perspectives with diabetes care services, whereas other patient satisfaction questionnaires measure selected aspects of diabetes care [16] such as evaluation of satisfaction with oral medication [27]. Interestingly, there is one other generic tool measuring patient satisfaction with diabetes disease management. However, it has more items (73 items) which assesses patients’ satisfaction with the structure and process of meetings, personal time commitment, the level of physical activity, and the personal nutrition requirements needed to maintain their health. The second strength is that the PSQ is a valid tool which was constructed by systematic review [4] and tested in two countries in the Association of Southeast Asian Nations (ASEAN). Pharmacy practice varies in ASEAN countries. Some countries involve pharmacists in direct patient care, but most countries do not [28]. So, this tool may help pharmacists to enhance the quality of diabetes management and encourage pharmacists to become more involved in diabetes care in the ASEAN context.

When considering the model fit indicators of the Thai and Lao PSQs, both models showed an acceptable Model chi-square and met the criteria for CFI and SRMR. Even though the GFI and RMSEA of the Lao model did not reach the standard, these fit indices are declining in usage and the SRMR and CFI are directly comparable [22]. The CFA showed differences among the two versions. The PSQ Thai version showed five factors while the PSQ Lao version showed three factors. So this might explain the low scores in dimension 4, Competency of Pharmacists. Thus, the dimension of Competency of Pharmacists was combined with dimension 5, Communication with Providers. An interesting point of difference was that the satisfaction score for the annual health checkup was low in the Standard of Service dimension in Lao PDR. The DIF showed differences between the two versions in the Standard of Service including health checkup, annual health checkup and health insurance. In Thailand, health checkups and annual health checkups were provided to all Thai people by all national health insurance schemes [29]. In Lao PDR, there was a health screening project under the Thai Lao collaboration [30] but there were no routine services provided for Lao people. Another explanation of differences may be that the electronic health information system to support medical services in Lao PDR is still faced with difficulties [31], while Thailand has a successfully implemented electronic medical database [32]. With the limited national health insurance coverage in Lao PDR [6], most Lao diabetes patients pay out-of-pocket for the services and medications [7], while diabetes patients in Thailand do not pay for their services and medication which is covered by national health insurance.

Thai pharmacists are already involved with diabetes care through patient counseling [33,34], pharmaceutical care plans [35], and home visits [36]. Various studies demonstrate good clinical outcomes for diabetes patients [34–36]. However, there were no home visit services nor pharmacists’ involvement with diabetes patients in Lao PDR. Thus, the difference between countries is a limitation for applying a valid and reliable tool to assess patient satisfaction in different countries [16].

The similarities in pharmacist roles include advocating health promotion, and providing information on healthy diets, physical activity, and compliance. However, there are differences in practices involving multidisciplinary teams on wards and in clinics which Thai pharmacists have been involved with. In contrast, there are limited roles for Lao pharmacists in clinics and hospitals, and a limited scope of advanced clinical pharmacy practice. This can be explained by better educational support in Thailand. PharmD curricula are implemented in all 19 universities, with students completing at least 2,000 practice hours. There is also a 4-year residency program for specialty pharmacists. Lao PDR has only one university that provides a 5-year curriculum with 7 months of practice experience [37]. At the present, Lao graduates are not accepted by the physicians in Lao PDR in terms of patient care collaboration and pharmacists have more limited relationships with patients than do physicians and nurses [38].

The PSQ Thai version item S6 (Fig 1), which is about follow-up services did not fit to its dimension (<0.5), Diabetes Service, but it did fit in the Competency of Providers dimension. This demonstrated that Thai diabetes patients had a perception of good care that was more from patient-provider interactions than the diabetes care system. This may be explained by a study on attitudes of Thai diabetes patients toward the health care team that found having a favorable attitude toward one’s physician, patients’ perceived compassion and understanding from one’s physician, or feeling as though the physician was an integral member of the diabetes care team, could encourage participants to take medication [39].

There were limitations to this study. The questionnaire was designed to be completed by patients; however, some patients were old and had limited time to respond to the PSQ, especially in Lao settings. So face to face interviews were mostly used in these patients. The PSQ is a tool to assess diabetes management with pharmacist involvement rather than overall satisfaction with diabetes treatment, so this may limit generalizability in practices without pharmacist participation. However, the Lao PSQ with three factors, including Standard of Services, Diabetes Service, and Satisfaction with Providers, could be used and reduce this limitation.

## Conclusions

The PSQ in both the Thai and Lao versions was found to be valid and reliable for the assessment of patient satisfaction with diabetes management. The five-dimension PSQ is recommended to evaluate patients’ perspectives on diabetes care services with pharmacist involvement, and the three-dimension PSQ is for diabetes care services without pharmacist involvement. As with all questionnaires, continuous testing and refinement are necessary.

## Supporting information

S1 File Patient satisfaction questionnaire for diabetes management (English version). (DOCX)
