# Supplementary material for: Development of a patient satisfaction questionnaire (PSQ) for diabetes management in Thailand and Lao PDR
Source: PLoS One. 2024 Mar 7;19(3):e0300052. doi: 10.1371/journal.pone.0300052 (PMC10919862; doi:10.1371/journal.pone.0300052)
Supplement: S1 File — (DOCX) [file pone.0300052.s001.docx]

**Satisfaction of diabetes patients with diabetes management program**

This questionnaire was developed to be a standard tool to evaluate a diabetes management program. There are two parts: demographic data, and a 20-item satisfaction survey with 5 dimensions.

**Suggestions for answering the questionnaire:** put an X next to the choice that best represents you or your opinion.

**Part 1: demographic data**

1. Gender ( ) 1.male ( ) 2.female

2. Date of Birth……………………………

3. Career

( ) 1. Government employee ( ) 2. Employee ( ) 3. Commercial

( ) 4. Farmer ( ) 5. Retired ( ) 6. Others, please specify…………

4.Education

( ) 1. No education ( ) 2. Primary school ( ) 3. Secondary school

( ) 4. High school ( ) 5. Associate’s degree ( ) 6. Bachelor’s degree

( ) 7. Graduate degree ( ) 8. Others, please specify…………

5. Average income per month in Baht (include all income)

( ) 1. < 5,000 ( ) 2. 5,001-10,000 ( ) 3. 10,001-15,000

( ) 4. 15,001-20,000 ( ) 5. > 20,001

6. Marital status

( ) 1. Single ( ) 2. Married ( ) 3. Widowed ( ) 4. Divorced ( ) 5. Separated

7. Number of people living at your residence: ........................................people

7.1 Please specify relationship ( ) Parents ( ) Grandparents ( ) Siblings

( ) Children ( ) Grandchildren ( ) Others, please specify………………

8. Distance between home and a primary care unit that you are receiving diabetes care service ................................kilometers

8.1 The name of your primary care unit......................................................

10. How long have you had diabetes? ………………………………. months……………………………….years

11. Do you have any co-morbidities?

( ) 1. No ( ) 2. Yes (specify, could be more than 1 disease) ..............................................

12. Information sources about diabetes that you have received within the prior of 3 months (Please mark all that apply)

( ) 1. Healthcare providers (physician/nurse/pharmacist/dietitian) ( ) 2. Radio/TV

( ) 3. Social media (Facebook, Line, Twitter…) ( ) 4. Friends/cousins

( ) 5. Others, please specify............................

13. Type of health insurance (Please mark all that apply)

( ) 1. Universal health coverage ( ) 2. Civil servant

( ) 3. Social security ( ) 4. Private insurance

( ) 5. Other, please specify............................

**Part 2: Satisfaction with diabetes management**

Each item measures the level of your satisfaction with your diabetes management. Please put an X in the appropriate column for each number.

**5 = highly satisfied 4 = satisfied 3 = uncertain 2= dissatisfied 1 = very dissatisfied**

**n/a = not applicable**

| No | Item | Satisfaction level | | | | | |
| --- | --- | --- | --- | --- | --- | --- | --- |
|  |  | 5 | 4 | 3 | 2 | 1 | n/a |
| Standard of Services | | | | | | | |
| 1 | Are you satisfied with having the diabetes services separated from other services? |  |  |  |  |  |  |
| 2 | How satisfied are you with the standard of health check at the beginning of every visit? (e.g., BP measurement, body weight, blood sugar) |  |  |  |  |  |  |
| 3 | How satisfied are you with the standard annual health check that you received? (e.g., retina, kidney, foot, heart) |  |  |  |  |  |  |
| 4 | How satisfied are you with the services received that were covered by your health insurance. |  |  |  |  |  |  |
| Type of Services | | | | | | | |
| 5 | How satisfied are you with the information that you have received? |  |  |  |  |  |  |
| 6 | How satisfied are you with the regular follow-up plan for diabetes? |  |  |  |  |  |  |
| 7 | How satisfied are you with the home visit done by the primary care unit? |  |  |  |  |  |  |
| 8 | How satisfied are you with the data collecting system of the primary care unit, such as satisfaction questionnaire for the services provided? |  |  |  |  |  |  |
| Competency of Providers | | | | | | | |
| 9 | How satisfied are you with the providers’ knowledge of your medical and treatment history? |  |  |  |  |  |  |
| 10 | How satisfied are you with the competency of the doctors to provide useful information on your diabetes selfcare? |  |  |  |  |  |  |
| 11 | How satisfied are you with the team that created your personalized treatment plan? |  |  |  |  |  |  |
| 12 | How satisfied are you with the amount of time the team spent with you? |  |  |  |  |  |  |
| Competency of Pharmacists | | | | | | | |
| 13 | How satisfied are you with the team’s understanding of your treatment history and medicines? |  |  |  |  |  |  |
| 14 | How satisfied are you with the competency of the pharmacists to provide useful information on the proper use of your diabetes medicines? |  |  |  |  |  |  |
| 15 | How satisfied are you with the pharmacist in preparing a personalized care plan for the taking of your medicines? |  |  |  |  |  |  |
| 16 | Are you satisfied with the amount of time the pharmacist spends with you for the care of your diabetes? |  |  |  |  |  |  |
| Communication with Providers | | | | | | | |
| 17 | Are you satisfied that the healthcare providers listen to you and are open to answering your questions? |  |  |  |  |  |  |
| 18 | Are you satisfied with the friendliness of your providers. |  |  |  |  |  |  |
| 19 | Are satisfied that the providers asked you to take part in the treatment plan? |  |  |  |  |  |  |
| 20 | Are satisfied with the simplicity of communication on your treatment plan? |  |  |  |  |  |  |
